# The *Verticillium dahliae* Spt-Ada-Gcn5 Acetyltransferase Complex Subunit Ada1 Is Essential for Conidia and Microsclerotia Production and Contributes to Virulence

**DOI:** 10.3389/fmicb.2022.852571

**Published:** 2022-02-23

**Authors:** Qi Geng, Huan Li, Dan Wang, Ruo-Cheng Sheng, He Zhu, Steven J. Klosterman, Krishna V. Subbarao, Jie-Yin Chen, Feng-Mao Chen, Dan-Dan Zhang

**Affiliations:** ^1^Co-Innovation Center for Sustainable Forestry in Southern China, Nanjing Forestry University, Nanjing, China; ^2^Team of Crop Verticillium Wilt, State Key Laboratory for Biology of Plant Diseases and Insect Pests, Institute of Plant Protection, Chinese Academy of Agricultural Sciences, Beijing, China; ^3^National Cotton Industry Technology System Liaohe Comprehensive Experimental Station, The Cotton Research Center of Liaoning Academy of Agricultural Sciences, Liaoning Provincial Institute of Economic Crops, Liaoyang, China; ^4^United States Department of Agriculture, Agricultural Research Service, Crop Improvement and Protection Research Unit, Salinas, CA, United States; ^5^Department of Plant Pathology, c/o U.S. Agricultural Research Station, University of California, Davis, Salinas, CA, United States

**Keywords:** *Verticillium dahliae*, SAGA complex, Ada1 subunit, melanin biosynthesis, virulence, transcriptional regulatory function

## Abstract

*Verticillium dahliae* is a destructive soil-borne pathogen of many economically important dicots. The genetics of pathogenesis in *V. dahliae* has been extensively studied. Spt-Ada-Gcn5 acetyltransferase complex (SAGA) is an ATP-independent multifunctional chromatin remodeling complex that contributes to diverse transcriptional regulatory functions. As members of the core module in the SAGA complex in *Saccharomyces cerevisiae*, Ada1, together with Spt7 and Spt20, play an important role in maintaining the integrity of the complex. In this study, we identified homologs of the SAGA complex in *V. dahliae* and found that deletion of the *Ada1* subunit (*VdAda1*) causes severe defects in the formation of conidia and microsclerotia, and in melanin biosynthesis and virulence. The effect of VdAda1 on histone acetylation in *V. dahliae* was confirmed by western blot analysis. The deletion of *VdAda1* resulted in genome-wide alteration of the *V. dahliae* transcriptome, including genes encoding transcription factors and secreted proteins, suggesting its prominent role in the regulation of transcription and virulence. Overall, we demonstrated that VdAda1, a member of the SAGA complex, modulates multiple physiological processes by regulating global gene expression that impinge on virulence and survival in *V. dahliae*.

## Introduction

*Verticillium dahliae* is a soilborne fungus that causes vascular wilt on more than 200 plant species and causes extensive economic losses worldwide (Klosterman et al., 2009). The disease cycle begins with *V. dahliae* microsclerotia germinating under favorable conditions, the emerging hyphae then penetrating plants through the roots and extensively colonizing the xylem vessels of susceptible hosts. In the vascular tissues, hyphae breach vessel end walls and conidia are produced within the xylem (Fradin and Thomma, 2006; Vallad and Subbarao, 2008). As the disease progresses, the water conducting xylem vessels become occluded, leading to wilting symptoms and in some cases death (Fradin and Thomma, 2006; Klosterman et al., 2009). Microsclerotia produced in the dead tissues are returned to the soil along with the plant debris to serve as the primary inoculum for subsequent crops to initiate new infection cycles (Wilhelm, 1955; Klimes et al., 2015). Methods of managing *V. dahliae* post-colonization of the xylem are unavailable, and the production of microsclerotia, which can survive over 10 years in the soil, also make *V. dahliae* difficult to control (Wilhelm, 1955; Johnson et al., 1980; Fradin and Thomma, 2006; Klosterman et al., 2009).

Transcription co-activator complexes play important regulatory roles in the virulence of plant pathogenic fungi (Rösler et al., 2016; Yang et al., 2020) by specifically regulating the chromatin landscape near the promoter. This process is necessary for efficient PIC (pre-initiation complex) assembly (Bhaumik, 2011), including the loading of RNA polymerase II (Pol II) and general transcription factors (GTFs) (Helmlinger and Tora, 2017). Spt-Ada-Gcn5 acetyltransferase complex (SAGA) is one of the first complexes shown to have co-activator activity in eukaryotes, and possesses both acetyltransferase and deubiquitinase activities that modify histones and activate transcription by recruiting the TATA-binding protein (TBP) to promoters (Roberts and Winston, 1997; Bhaumik and Green, 2001). The SAGA complex is highly conserved in eukaryotes such as *Schizosaccharomyces pombe*, *Fusarium fujikuroi*, *Drosophila melanogaster*, *Mus musculus*, and *Homo sapiens* (Huisinga and Pugh, 2004).

Four distinct functional modules have been elucidated in the SAGA complex of the model yeast *S. cerevisiae* (Sugiura et al., 1998; Cheon et al., 2020). One of these, referred to as the HAT module, includes proteins Gcn5, Ada2, Ada3, and Sgf29. Members of the HAT module have preference for acetylation of histone components such as H3K14 (Kuo et al., 1996). Gcn5 can also acetylate H3K9, H3K18, H3K23, H3K27, H3K36, and additional lysines found in histones H2B and H4 (Brownell et al., 1996; Kuo et al., 1996; Grant et al., 1999; Morris et al., 2007). There is evidence that Ada2 enhances catalytic HAT activity, while Ada3 facilitates nucleosomal acetylation and expands the lysine specificity of Gcn5 in the complex (Balasubramanian et al., 2002). The conserved Sgf29 subunit recognizes H3K4 trimethylation, and activates transcription through recruitment of TBP to promoters (Bian et al., 2011; Shukla et al., 2012). Although originally discovered and characterized as a HAT complex (Grant et al., 1997), SAGA was later found to contain a second catalytic module known as the DUB module, which includes Ubp8, Sus1, Sgf73, and Sgf11. Ubp8 deubiquitinates H2B to promote transcription elongation (Wyce et al., 2007; Cheon et al., 2020) and associates with SAGA through Sgf11 and Sgf73, with Sus1 as an additional subunit (Lee et al., 2005, 2009; Durairaj et al., 2014). The core module of the SAGA complex comprises the most subunits, including Taf5, Taf6, Taf9, Taf10, Taf12, Spt7, Spt20, Spt3, Spt8, and Ada1. In this module, Ada1, Spt7, and Spt20 are necessary to ensure the structural integrity of the SAGA complex (Grant et al., 1997; Sterner et al., 1999). Spt3 and Spt8 activate transcription by the recruitment of TBP to particular promoters *in vivo* (Laprade et al., 2007; Papai et al., 2020). The TF-binding module comprises a single subunit Tra1 and forms a single lobe in SAGA. Tra1 was confirmed to physically interact with transcriptional activators owing to a HEAT (Huntingtin, elongation factor 3, PR65/A, and TOR) domain (Knutson and Hahn, 2011; Sharov et al., 2017). The interaction between Tra1 and the main lobe relies on a narrow hinge, which provides structural flexibility for regulatory functions (Sharov et al., 2017).

The SAGA complex plays an important role in the virulence of plant pathogenic fungi. In *Aspergillus flavus*, an opportunistic fungal pathogen of oil crops, AflGcnE (Gnc5) is crucial for regulating developmental processes affecting growth, sporulation, sclerotial formation, stress resistance, seed colonization, and aflatoxin biosynthesis (Lan et al., 2016). In *F. fujikuroi*, Gcn5 and its interacting partners, Ada2 and Ada3, regulate growth and asexual reproduction as well as secondary metabolism due to the manifold acetylation targets at H3 (Rösler et al., 2016). Interestingly, FgGcn5 is a direct antifungal target of the compound Phenazine-1-carboxamide (PCN), which is secreted by the bacterium, *Pseudomonas piscium* ZJU60, and causes defects in histone acetylation, fungal growth, virulence, and mycotoxin biosynthesis in *F. graminearum* (Chen et al., 2018). Spt3 and Spt8 are also essential for conidiation, sexual reproduction, and virulence, and play an important role in pigment formation in *F. graminearum* (Gao et al., 2014). In addition, *MoUbp8* is required for conidiation, melanin deposition, appressorial development and virulence, and is involved in carbon catabolite repression in *Magnaporthe oryzae* (Yang et al., 2020). Among the SAGA complex members, there is little known on the role of Ada1 in plant fungal pathogens.

Ada1, a subunit of the core module in the SAGA complex, was originally isolated based on toxicity in yeast cells of the chimeric activator, GAL4-VP16 (Berger et al., 1992). The *Ada1* mutants exhibit defects in transcription for specific genes and its phenotypic defects are broader than those found in *Ada2*, *Ada3*, and *Gcn5* mutants (Horiuchi et al., 1997). In contrast to the modest effects on structure from the loss of either Gcn5, Spt3, or Spt8, Ada1 is critical to the structural integrity of the SAGA complex (Sterner et al., 1999). The SAGA complex without Ada1 lacks the HAT module (Gcn5, Ada2, Ada3, and Sgf29), Taf12, Spt3, and Tra1 (Wu and Winston, 2002; Wu et al., 2004). Ada1 also functions in delimiting the boundaries of gene silencing in chromatin and this function was linked to proteasome processes (Kamata et al., 2016). In addition, Ada1 is also involved in repairing radiation damage (Game et al., 2005), regulation of gene expression in the yeast metabolic cycle (Sánchez-Gaya et al., 2018), and nuclear localization of Ubp8 subunit (Nuño-Cabanes et al., 2020).

In *V. dahliae*, dozens of transcription factors participate in the regulation of vegetative growth, melanin biosynthesis, and virulence (Klimes et al., 2015; Luo et al., 2016; Wang et al., 2018). Beside transcription factors, post-translational histone modifications (PTMs) are also important epigenetic mechanisms for regulating gene expression. *VdmilR1* targets *VdHy1*, a gene essential for virulence, for transcriptional repression through increased histone H3K9 methylation of *VdHy1* (Jin et al., 2019). *VdBre1*, a ubiquitin ligase (E3) enzyme which modifies H2B, plays crucial roles in cotton infection and virulence by globally regulating lipid metabolism and secondary metabolism (Wang et al., 2021). In addition, components of chromatin remodeling complex ISW2 – VdDpb4 and its interacting protein, VdIsw2, are essential for *V. dahliae* responses to ROS stress and VdDpb4 is required for VdIsw2-dependent transcriptional regulation of gene expression (Wang S. et al., 2020).

Though the SAGA complex plays a role in the pathogenicity of plant pathogens (Rösler et al., 2016; Chen et al., 2018; Yang et al., 2020), the complex has not been studied in *V. dahliae*. The main objectives of the current study were to: (1) identify the composition of the SAGA complex in *V. dahliae*; (2) investigate the role of the SAGA subunit Ada1 in the virulence of *V. dahliae*; and (3) elucidate the regulatory role of Ada1 in *V. dahliae*.

## Results

### Composition of the Spt-Ada-Gcn5 Acetyltransferase Complex in *V. dahliae*

**FIGURE 1:**
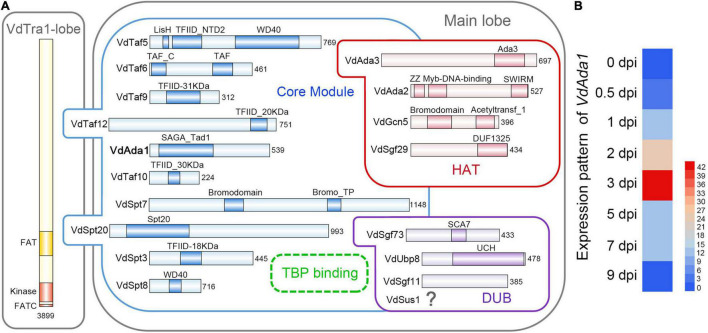
Identification of the SAGA complex and Ada1 subunit in *Verticillium dahliae*. **(A)** Subunit and domain organization of the SAGA complex in *V. dahliae*. SAGA subunits are organized into the Tra1 lobe and the main lobe. The main lobe is divided into enzymatic HAT and DUB modules and a core module. The dotted circle near Spt3 and Spt8 indicates the TBP-binding site, where TBP is recruited. The numbers behind each subunit represent the number of amino acids. Figure design adapted from Cheon et al. (2020) and Papai et al. (2020) **(B)** Expression pattern analysis of *VdAda1*. The level of *VdAda1* transcription was detected by reverse transcription-quantitative PCR during colonization on cotton at 0, 0.5, 1, 2, 3, 5, and 7 days post-inoculation (dpi). The expression level of *VdAda1* at 0 dpi was set to 1.

To identify putative members of the SAGA complex in *V. dahliae* isolate AT13, we first searched the *V. dahliae* genome sequence by BlastP using protein sequences of the known *S. cerevisiae* SAGA complex members as queries. Using this approach, nearly all SAGA homologs in *V. dahliae* were identified except Sus1, one of the subunits belonging to DUB module. Among the 18 subunits identified, Ada1 (FFUJ_07552) and Sgf11 (FFUJ_06279) were identified as present in *F. fujikuroi* (Rösler et al., 2016) since there were no homologs in *S. cerevisiae* (Figure 1A).

Given the importance of Ada1 in SAGA complex, the function of VdAda1 in *V. dahliae* was further explored. The full *VdAda1* (VDAG_08660) gene is 1860 bp in length, encoding a protein with 539 amino acids with a conserved SAGA_Tad domain. The predicted protein sequence of VdAda1 shares 75.3% identity with the sequence of Ada1 from *F. fujikuroi* (Figure 1A).

Although a large number of studies have shown that the SAGA complex and Ada1 homologs play an important role in multiple physiological processes of fungi, the potential role of VdAda1 in *V. dahliae*-plant interactions has not been explored. To assess the relationship between *VdAda1* expression and virulence in *V. dahliae*, the expression pattern of *VdAda1* was monitored by reverse transcription-quantitative PCR (RT-qPCR) at different times following inoculation. The results indicate that the expression of *VdAda1* was induced by cotton, especially at 3 dpi (Figure 1B), suggesting that *VdAda1* may play an important role in the virulence of *V. dahliae*.

### *VdAda1* Is Essential for the Vegetative Growth and Production of Conidia

**FIGURE 2:**
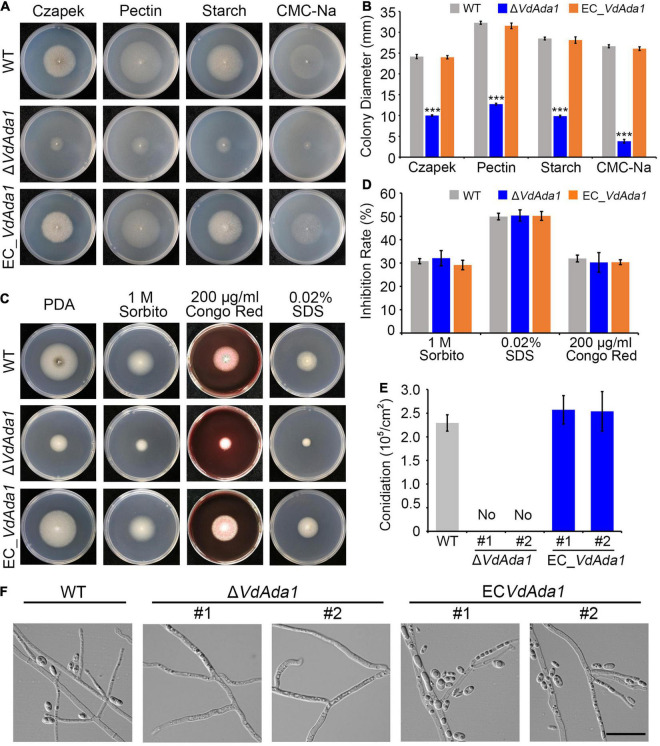
*VdAda1* in *Verticillium dahliae* is necessary for conidia production and normal growth rate, but not for cell wall integrity and osmotic stress responses. **(A)** Colony morphology of the wild type strain, Δ*VdAda1*, and EC_*VdAda1* strains on Czapek medium containing different four carbon sources. **(B)** Colony diameter (mm) of each strain grown on different medium. Values shown are the average of three colony diameters. Error bars represent the standard deviation calculated from three replicate experiments, and a triple asterisk indicates significant differences (*P* < 0.001) in colony diameter of Δ*VdAda1* compared with the wild type using one-way analysis of variance (ANOVA). **(C)** Colony appearances of the wild type, Δ*VdAda1*, and EC_*VdAda1* strains cultured on Czapek medium containing 1 M sorbitol, 200 μg/ml Congo red, and 0.02% SDS at 25°C for 7 days. **(D)** Sensitivity of the wild type, Δ*VdAda1*, and EC_*VdAda1* strains to different cell wall stress chemicals. Values shown are the average from three repeated experiments and the data were analyzed using one-way analysis of variance (ANOVA). Error bars represent the standard deviation of three replicate experiments. **(E)** Quantification of conidial production. After the strains were grown on Czapek medium for 7 days, three 7 mm-diameter plugs of the fungal colony were collected by a hole puncher and shaken in 3 mL of sterile water, and the number of conidia were counted by a hemocytometer. The #1 and #2 indicate two independent deletion mutants or complemented transformants. Values shown are the average from three repeated experiments and the data were analyzed using one-way analysis of variance (ANOVA). Error bars represent the standard deviation calculated from three replicate experiments. **(F)** Observations of hyphae and production of conidia by the wild type, Δ*VdAda1*, and EC_*VdAda1* strains grown on hydrophobic cover slips for 5 days using a differential interference contrast microscope. Scale bar = 20 μm.

To further understand the role of Ada1 subunit during *V. dahliae* – host interactions, we generated deletion mutants of Ada1 subunit (Δ*VdAda1*) and complemented transformants (EC_*VdAda1*) of the Δ*VdAda1* mutant (Supplementary Figure 1). Since *Ada1* homologs in yeast are involved in vegetative growth (Horiuchi et al., 1997; Sterner et al., 1999), we assessed the growth of Δ*VdAda1* strain in comparison with the wild type (AT13) and EC_*VdAda1* strains on different carbon sources. The Δ*VdAda1* strain grew significantly slower than the wild type strain on medium containing four different carbon sources (sucrose, starch, pectin, and CMC-Na) (Figures 2A,B). In fact, the colony diameter of the Δ*VdAda1* strain was less than half of that of AT13, whereas the defect was remedied in the EC_*VdAda1* strain (Figures 2A,B). We also examined the sensitivity of Δ*VdAda1* strain to cell wall integrity and osmotic stressors including sorbitol, Congo red, and sodium dodecyl sulfate (SDS). The sensitivity of Δ*VdAda1* to these stress factors was not different from that of the wild type strain (Figure 2C) nor was there a statistically significant difference between any of the strains (Figure 2D), suggesting that *VdAda1* is not involved in the regulation of cell wall integrity and osmotic stress response in *V. dahliae*.

Analyses of conidia harvested from Czapek plates at 7 days post incubation revealed that the Δ*VdAda1* strain did not produce conidia while the EC_*VdAda1* strain produced as many conidia as the wild type (Figure 2E). After placing each strain onto hydrophobic cover slips, we observed the hyphae and conidia on cover slips under a fluorescence microscope at 5 dpi and found that only hyphae but no conidia could be observed in the Δ*VdAda1* strain. In the wild type and EC_*VdAda1* strains, we observed both hyphal growth and production of conidia (Figure 2F). However, the morphology of hyphae in Δ*VdAda1* strain was not different from that of wild type and EC_*VdAda1* strain (Figure 2F). Collectively, these data demonstrate that *VdAda1* is not involved in the regulation of cell wall integrity but is necessary for vegetative growth and conidia production.

### Effect of *VdAda1* on the Expression of Genes Involved in Melanin Synthesis and Microsclerotia Formation

**FIGURE 3:**
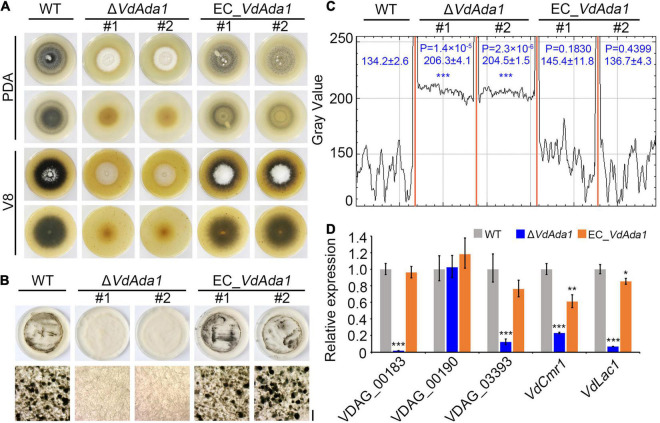
*VdAda1* is involved in melanin synthesis and microsclerotia formation in *Verticillium dahliae*. **(A)** Colony morphology of the wild type (AT13), Δ*VdAda1*, and EC_*VdAda1* strains on PDA and V8 medium plates at 2 weeks post-incubation. Δ*VdAda1* strains show reduced melanin production, and mutant complementation restored wild type morphology. **(B)** The conidial suspensions of the wild type, Δ*VdAda1*, and EC_*VdAda1* strains were adjusted to 1 × 10^6^ propagules/ml and coated on BMM medium overlaid with cellophane layer. After 2 weeks incubation at 25°C, the appearance of microsclerotia on the cellophane layer were observed and photographed by stereo microscopy (Below). Scale bar = 20 μm **(C)** Gray-value curves were obtained by scanning microscopy photographs of microsclerotia in **(B)** using ImageJ software. The numbers indicate the range of gray values of each strain. The asterisks show significant differences (***P < 0.001) compared with the wild type using one-way analysis of variance (ANOVA). **(D)** *VdAda1* regulates the expression of melanin-associated genes. RT-qPCR was used to measure expression levels of melanin-associated genes in the wild type, Δ*VdAda1*, and EC_*VdAda1* strains cultured on BMM medium overlaid with a cellophane membrane for 2 weeks. The *V. dahliae* elongation factor 1-α (*VDEF-1*α) was used as an endogenous control for RT-qPCR. Error bars represent the standard deviation calculated from three replicate experiments, and the asterisks indicate significant treatment differences (***P < 0.001, **P < 0.01, and *P < 0.05, respectively) in expression compared with the wild type using one-way analysis of variance (ANOVA).

Fungal melanins can enhance resistance to environmental stresses (Wang et al., 2018; Eisenman et al., 2020) and may also modulate immune responses of the host (Chai et al., 2010; Volling et al., 2011). Though DHN melanin itself is not a virulence factor in *V. dahliae* (Wang et al., 2018), production of melanin and microsclerotia are tightly linked in *V. dahliae* and some of the pathways that regulate melanin biosynthesis also influence virulence in *V. dahliae* (Fan et al., 2017). To investigate the effect of *VdAda1* on melanin synthesis and microsclerotia formation in *V. dahliae*, the wild type, Δ*VdAda1*, and EC_*VdAda1* strains were incubated on PDA medium and V8 medium at 25°C for 2 weeks. The Δ*VdAda1* strain failed to produce melanin on PDA medium or on V8 medium while the wild type strain and EC_*VdAda1* strain were clearly melanized (Figure 3A). The conidial suspension of AT13 strain and EC_*VdAda1* strain together with protoplasts of Δ*VdAda1* strain were adjusted to approximately 1 × 10^6^ propagules ml^–1^ and coated on microsclerotia induction medium (BMM medium) overlaid on a cellophane layer. Cultures of these strains at 25°C for 2 weeks followed by microscopy revealed that the AT13 strain formed many melanized microsclerotia, while the Δ*VdAda1* strain did not produce melanin or microsclerotia (Figure 3B). Melanized microsclerotial production was restored in EC_*VdAda1* strain (Figure 3B). Analyses of gray-scale curves using Image J software verified the defect in melanin synthesis in the Δ*VdAda1* strain (Figure 3C).

Numerous genes in *V. dahliae* have been characterized for their roles in melanin synthesis and microsclerotia formation (Klimes et al., 2015; Zhang et al., 2017; Wang et al., 2018). The expression of melanin biosynthetic genes is tightly correlated with microsclerotia development (Duressa et al., 2013). RT-qPCR expression analyses of five genes involved in melanin synthesis in *V. dahliae* revealed that the majority of these genes were significantly downregulated in the Δ*VdAda1* strain and that their expression in complemented strains was restored to the wild type level (Figure 3D). These results suggested that *VdAda1* plays an essential role in melanin synthesis and microsclerotia formation in *V. dahliae* by regulating the transcription of genes involved in these processes.

### *VdAda1* Has a Strong Impact on the Virulence of *V. dahliae*

**FIGURE 4:**
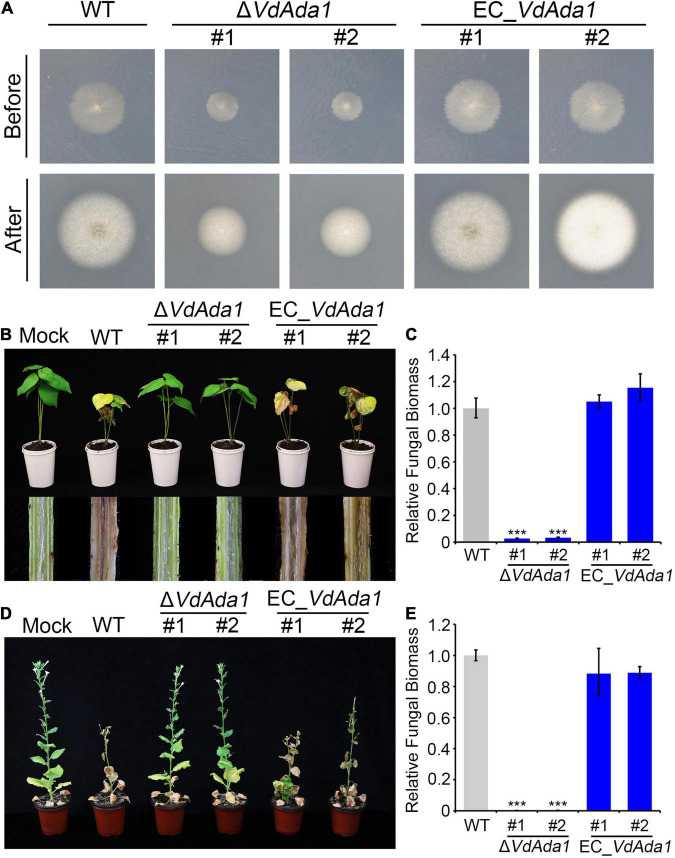
*VdAda1* is required for the full virulence in *Verticillium dahliae*. **(A)** The first row shows colonies of the wild type, Δ*VdAda1*, and EC_*VdAda1* strains grown on minimal medium (MM) overlaid with a cellophane layer at 3 dpi (before). Photographs in the second row were taken at 5 days after removal of the cellophane layer (after). **(B)** Phenotypes of 2-week-old cotton seedlings inoculated with agar plug (Mock), hyphae from PDA agar cultures of wild type *V. dahliae* strain AT13, the Δ*VdAda1* mutant strains, and complemented strains. The virulence phenotypes were photographed at 14 dpi. The disease symptoms are shown at the top, and the discoloration of the inoculation shoot longitudinal sections is shown at the bottom. **(C)** The fungal biomass of Δ*VdAda1* strains, and EC_*VdAda1* strains on cotton were detected by quantitative PCR (qPCR). *V. dahliae* elongation factor 1-α (*VdEF-1*α) was used to quantify fungal colonization. The cotton *18S* gene was used as endogenous plant reference gene. Error bars represent the standard deviation calculated from three replicate experiments compared with the wild type. ***, significant differences (*P* < 0.001) using one-way analysis of variance (ANOVA). **(D)** Phenotypes of 5-week-old *N. benthamiana* plants inoculated with agar plug (Mock), hyphae from PDA agar cultures of wild type AT13, Δ*VdAda1* mutant, and complemented strains. The virulence phenotypes were photographed at 21 dpi. **(E)** The fungal biomass of Δ*VdAda1* strains, and EC_*VdAda1* strains on *N. benthamiana* were detected by qPCR. *V. dahliae* elongation factor 1-α (*VdEF-1*α) was used to quantify fungal colonization. The *N. benthamiana EF-1*α was used as endogenous plant reference gene. Error bars represent the standard deviation calculated from three replicate experiments, ***, significant differences (*P* < 0.001) compared with the wild type using one-way analysis of variance (ANOVA).

To investigate the role of *VdAda1* during the initial infection of *V. dahliae*, we analyzed the penetration abilities of *VdAda1* by incubating wild type, Δ*VdAda1*, and *VdAda1* complemented transformants on a cellophane membrane overlaid on minimal medium. At 3 dpi, the cellophane membrane was removed and hyphal penetration into the cellophane membrane was examined at 8 dpi (Figure 4A). The results in Figure 4A suggest that *VdAda1* is not involved in penetration. To analyze the role of *VdAda1* on virulence, seedlings of cotton (*Gossypium hirsutum* cv. Junmian 1) and *Nicotiana benthamiana* were inoculated with hyphae from cultures of the wild type, Δ*VdAda1*, and EC_*VdAda1* strains on PDA. The results revealed that the Δ*VdAda1* strain had lost the ability to infect cotton (Figure 4B). There was also a significant reduction in the biomass of the Δ*VdAda1* strain in inoculated cotton plants (Figure 4C). The Δ*VdAda1* strain was also non-pathogenic on *N. benthamiana* (Figure 4D). Virulence and fungal biomass measurements were restored in the *VdAda1* complemented strains (Figures 4B–E).

### VdAda1 Plays a Role as a Spt-Ada-Gcn5 Acetyltransferase Subunit That Acetylates Histone 3

**FIGURE 5:**
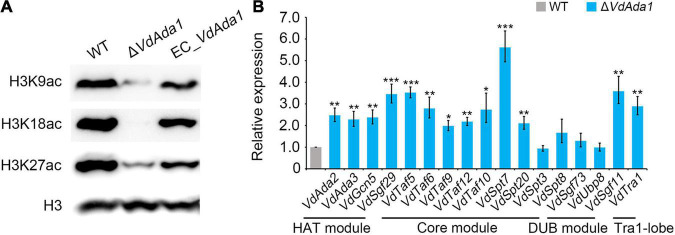
Influence of *VdAda1* on histone acetylation in *Verticillium dahliae*. **(A)** Different acetylation of histone 3 (H3) were detected in wild type, Δ*VdAda1*, and EC_*VdAda1* strains, using anti-H3K9ac, -H3K18ac, -H3K27ac antibodies. Hybridization with an anti-H3 C-terminus antibody served as a loading control. The molecular weight of the tested proteins was 17 kDa. **(B)** RT-qPCR was performed to detect the change of expression levels of all the subunits of the SAGA complex in Δ*VdAda1* strain compared with wild type strain. Error bars represent the standard deviation calculated from three replicate experiments, and the asterisks show significant differences (***P < 0.001; **P < 0.01; *P < 0.05) in expression compared with the wild type using one-way analysis of variance (ANOVA).

The SAGA complex primarily is described as an acetylation modifier of certain lysine residues on histones that regulates gene expression (Fischer et al., 2019). Ada1, as a member of the core module in the SAGA complex, plays an essential role in ensuring the structural integrity of the SAGA complex. In this study, several putatively relevant acetylation marks at histone 3 (H3) were analyzed at a global level. To investigate the involvement of Ada1 in acetylation of H3 in *V. dahliae*, the wild type, Δ*VdAda1*, EC_*VdAda1* strains were tested in western blot analyses using the anti-H3K9ac, -H3K18ac, -H3K27ac antibodies, which can detect acetyl modifications. H3K27 acetylation was significantly reduced in the deletion mutants compared to wild type strains (Figure 5A). H3K9 and H3K18 acetylation were nearly lost in the Δ*VdAda1* strain, while the acetylation levels in the EC_*VdAda1* strain was restored to the wild type levels (Figure 5A). We confirmed that *VdAda1* deletion resulted in the impaired ability to acetylate histone 3, and likely influenced gene expression.

To further study the mechanisms of how *VdAda1* affects histone 3 acetylation, we examined the transcript levels of other SAGA members in Δ*VdAda1* strain by RT-qPCR. Interestingly, the expression of almost all the SAGA subunits were induced after deletion of *VdAda1* (Figure 5B). The results revealed the important role of *VdAda1* in maintaining structural integrity of the SAGA complex, thus ensuring the ability to acetylate histone.

### Deletion of *VdAda1* Results in Genome-Wide Alteration of the *V. dahliae* Transcriptome

**FIGURE 6:**
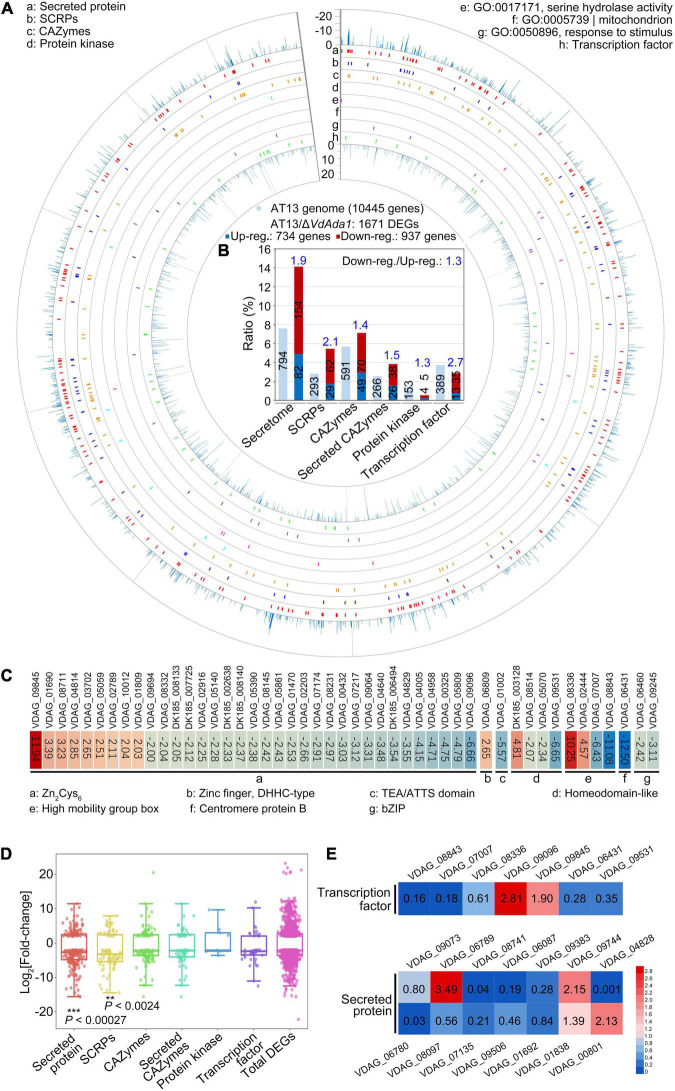
RNA-seq analysis reveals altered transcription in the Δ*VdAda1* strain of *Verticillium dahliae*. **(A)** Expression pattern of 1671 DEGs in the Δ*VdAda1* genome compared to the wild type strain. The DEGs of Δ*VdAda1* versus wild type with cutoff log_2_ ratio ≥2 and *P*-value < 0.01. **(B)** Bioinformatics analysis of potential virulence-related genes regulated by *VdAda1*. The numbers in the columns indicate the number of different types of genes in the AT13 genome or DEGs. The numbers in blue above the columns indicate the ratio between downregulated and upregulated genes. **(C)** Heat map of differentially expressed transcription factors. Colored blocks from blue to red represent downregulated to upregulated genes, respectively; the fold change is the value of the log_2_ ratio; the corresponding gene ID is at the top of the colored blocks, and the sequences of genes beginning with DK185 that was only found in strain AT13 but not in VdLs.17 reference genome were listed in Supplementary Table 3. **(D)** Fold-change range of DEGs regulated by *VdAda1*. The range of fold-change is displayed in the Box whisker plot. The central point in each box plot represents the median, the rectangle gives the interval between the 25 and 75% percentiles, and the whisker indicates the range. Significance of log_2_ fold-change of secreted proteins and SCRPs compared with the total DEGs was identified using Student’s *t*-test. **(E)** Expression level of selected genes encoding secreted proteins and transcription factors were detected in the Δ*VdAda1* strain compared with the expression level in wild type strain by RT-qPCR. The expression level of each gene in wild type strain was set to 1. The numbers inside the colored blocks represent the fold change of each gene.

To investigate the regulatory targets of *VdAda1* in *V. dahliae*, transcriptomes of Δ*VdAda1* and wild type strain AT13 were compared after 5 days of growth on Czapek medium. For these analyses, differentially expressed genes (DEGs) between the wild type and Δ*VdAda1* strains were considered significant with the cutoff log_2_ ratio ≥2 and *P*-value of <0.01. The RNA-seq analysis identified 1671 DEGs in total, including secreted proteins, small cysteine-rich proteins (SCRPs), CAZymes, protein kinase, transcription factors (TFs), and genes responsible for serine hydrolase activity, gene expressed in mitochondria and those involved in responses to stimuli (Figure 6A). GO annotation also indicated that DEGs mainly involved in the signal transduction, cellular response to stimulus, cell communication, response to stimulus, and signaling biological processes (Supplementary Figure 2). Among these DEGs, secreted proteins (secretome) accounted for the largest proportion (DEGs of secretome/1671 DEGs, 14.1%), followed by CAZymes (DEGs of CAZymes/1671 DEGs, 7.1%) and small cysteine-rich proteins (SCRPs, and DEGs of SCRPs/1671 DEGs, 5.4%). Of the 293 SCRPs encoded in AT13 genome, 91 genes (accounting for 31.1% DEGs of SCRPs/293) were differentially expressed, while 29.7% (DEGs of secretome/794 secreted proteins in DK185 genome) of the secreted proteins were differentially expressed, and thus were the two types of genes most significantly affected by the lack of *VdAda1* (Figure 6B). Furthermore, the ratio between downregulated and upregulated DEGs was obviously unbalanced (downregulated total DEGs/upregulated total DEGs, 1.3) (Figure 6B), indicating that *VdAda1* positively regulated expression of many genes. However, the downregulation of transcription factors in the Δ*VdAda1* strain were 2.7 times that of upregulated transcription factors (Figure 6B). The ratio of downregulated and upregulated secreted proteins was 1.9, while the ratio of downregulated and upregulated SCRPs was 2.1 (Figure 6B). The heat map further illustrated that particular transcription factors were significantly up or downregulated by *VdAda1*, and most of these belonged to the family of Zn_2_Cys_6_ TFs (Figure 6C). Moreover, the expression levels of the DEGs compared to the total DEGs of Δ*VdAda1* strain further confirmed that the expression levels of secreted proteins and SCRPs was different from the total DEGs, and an increased proportion was genes downregulated in the Δ*VdAda1* strain versus the wild type (Figure 6D). To confirm the role of *VdAda1* in regulating genes encoding secreted proteins and transcription factors, the expression of selected genes was quantified in the Δ*VdAda1* strain relative to the wild type by RT-qPCR. The results revealed that *VdAda1* showed a significant regulatory effect on the expression of these genes (Figure 6E). In summary, we confirmed that transcription factors and secreted proteins are targets regulated by VdAda1, thereby affecting multiple physiological functions, including virulence.

Kyoto Encyclopedia of Genes and Genomes (KEGG) annotations revealed that DEGs regulated by *VdAda1* were enriched in 169 pathways (Supplementary Table 2). Among these, multiple genes involved in carbon metabolism (ko:01200) were downregulated in the Δ*VdAda1* strain versus the wild type strain, and this may be related to the growth defect of Δ*VdAda1* strain (Figure 2 and Supplementary Figure 3). We also identified DEGs involved in starch and sucrose metabolism (ko:00500), which is speculated to be required for the degradation of plant cell walls (Supplementary Figure 4), suggesting that *VdAda1* regulates not only the expression of virulence-related genes but also the genes involved in growth and nutrient utilization.

## Discussion

In this study, *VdAda1* and other 17 SAGA subunits were identified in the *V. dahliae* genome database by using homologs from *S. cerevisiae* and *F. fujikuroi* as queries. Considering the importance of Ada1 in the SAGA complex, the role of *VdAda1* in conidiation, vegetative growth and virulence was explored. Furthermore, RNA-Seq analyses revealed multifaceted disturbances of *VdAda1* in regulating many pathways, including transcription factors and secreted proteins involved in growth and virulence. The deletion of *VdAda1* potentially led to the destruction of the structure and function of the SAGA complex, thereby impairing its ability to acetylate histone H3. This defect would globally disturb histone modifications which are necessary for the appropriate regulation of many genes in different pathways.

The first low-resolution 3D structure of the SAGA complex was derived from electron microscopy. In this model, the SAGA complex was divided into five domains and the critical core subunits (Ada1, Spt7, and Spt20) had been mapped to a central location within the complex (Wu et al., 2004). The latest high-resolution cryo-EM (electron cryomicroscopy) structures of the SAGA from *P. pastoris* and *S. cerevisiae* support these results (Papai et al., 2020; Wang H. B. et al., 2020). These studies divided the SAGA modules as follows: Tra1 module, core module (Spt3, Spt8, Spt7, TAFs, Ada1, and Spt20), HAT module (Gcn5, Ada2, Ada3, and Sgf29), and DUB module (Sgf73, Sgf11 Sus1, and Ubp8) (Papai et al., 2020; Wang H. B. et al., 2020). When we identified the composition of the SAGA in *V. dahliae* using protein sequences of known *S. cerevisiae* SAGA complex members as queries, we did not identify Ada1, Sgf11, and Sus1 homologs in *V. dahliae*. In *S. cerevisiae*, both Ada1 and Spt7 are responsible for integrity of the SAGA complex (Grant et al., 1997; Sterner et al., 1999; Wu and Winston, 2002). Fungal Spt7 proteins were confirmed to have two different evolutionary paths and are divided into yeast and filamentous fungal groups (Chen et al., 2018), so Ada1 may also have a similar evolutionary path such that the Ada1 homolog was not identified in *V. dahliae*. However, the predicted protein sequences of VdAda1 shared 75.3% identity with the sequences of Ada1 of *F. fujikuroi*, 70.3% identity with *A. fumigatus*, and 97.1% identity with *V. nonalfalfae*. The yeast SAGA DUB module is formed by the assembly of Ubp8 with Sgf11, Sus1, and the N-terminal residues of Sgf73 (Lee et al., 2009; Georgakopoulos et al., 2013; Helmlinger et al., 2021). The association of Ubp8 with SAGA is mediated by Sgf11 (Shukla et al., 2006), while Sus1 has been shown to be required for the H2B-deubiquitylation activity of DUB (Köhler et al., 2006). In several filamentous fungi, the absence of confirmed Sus1p or Sgf11p homologs (Georgakopoulos et al., 2013; Rösler et al., 2016), may explain why the Sgf11 homolog was identified in *F. fujikuroi* (Identity: 73%) but not in *S. cerevisiae*, and there were no apparent Sus1 homologs in *V. dahliae*.

Increasing numbers of studies have focused on the function of the SAGA complex in plant pathogens. The HAT module subunits Gcn5, Ada2 and Ada3, the DUB module subunit Ubp8, as well as the core module subunits Spt3 and Spt8 are reported to be involved in vegetative growth, conidiation and virulence (Gao et al., 2014; Rösler et al., 2016; Chen et al., 2018; Yang et al., 2020). In this study, compared with the wild-type strain and complemented transformants, the radial growth of *VdAda1* deletion mutant was significantly delayed (Figures 2A,B). The deletion of *VdAda1* lead to the loss of conidia production (Figures 2E,F). These results are consistent with previous studies, in which the absence of Ada1 caused more pronounced phenotypic defects than Ada2, Ada3, and Gcn5 mutants in yeast. More specifically, Ada1 mutant was auxotrophic for inositol (Horiuchi et al., 1997) and was unable to grow on media containing caffeine or the DNA synthesis inhibitor hydroxyurea (Sterner et al., 1999).

In dimorphic filamentous fungi, several SAGA subunits are also essential for stress responses (Gonzalez-Prieto et al., 2014; Chang et al., 2015). Gcn5 was confirmed to respond to cell wall stress induced by calcofluor white and Congo red in *A. flavus* (Lan et al., 2016). Deletion of Ubp8 also reduced the resistance of *M. oryzae* to not only osmotic stress but also cell wall stress (Yang et al., 2020). However, Spt3 and Spt8 are not involved in the regulation of cell wall integrity in *F. graminearum* (Gao et al., 2014). In our study, the sensitivity of the Δ*VdAda1* strain to osmotic stress induced by sorbitol, SDS and CR were not different from that of the wild-type strain or EC_*VdAda1* strain (Figures 2C,D), suggesting that SAGA members respond differently to environmental stresses.

The SAGA complex plays a critical role in the regulation of secondary metabolite (SM) biosynthesis. In *F. fujikuroi*, the expression of the known SM cluster genes was broadly affected by Gcn5 deletion (Rösler et al., 2016). In *A. flavus*, Gcn5 regulates aflatoxin production (Lan et al., 2016). In addition, Ubp8 was essential for melanin production in *M. oryzae* (Yang et al., 2020). To investigate the effect of *VdAda1* on melanin synthesis in *V. dahliae*, we compared the differences in the abilities of melanin synthesis and microsclerotia formation between the wild type and mutants. Noticeably, the albino Δ*VdAda1* mutant could not form melanized microsclerotia (Figures 3A–C). Furthermore, we quantified the expression levels of genes related to melanin. For example, VDAG_03393, encoding a scytalone dehydratase, is also involved in DHN (dihydroxynaphthalene melanin) melanin biosynthesis (Fan et al., 2017; Wang et al., 2018), 1,8-DHN-associated genes cluster including *VDAG_00183* (encoding a tetrahydroxynaphthalene reductase), *VDAG_00189* (*VdLac1*, encoding a laccase) and *VDAG_00190* (*VdPKS1*, encoding a polyketide synthase) regulated by *VdCmr1* (Wang et al., 2018). The expression of these genes was significantly decreased in the Δ*VdAda1* strain, except for *VdPKS1* (Figure 3D). In general, these results suggested the essential role of *VdAda1* in DHN melanin synthesis and microsclerotia formation. As for why the expression of VdPKS1 is not regulated by *VdAda1*, we hypothesize that polyketide synthases (PKSs) have diverse roles in secondary metabolite biosynthesis and in signaling (Eisenman and Casadevall, 2012; Kheder et al., 2012; Zhang et al., 2017), and VdPKS1 is probably regulated by other physiological processes, such as the conidial production and growth process (Zhang et al., 2017), and UV irradiation tolerance (Wang et al., 2018).

In the SAGA complex, the HAT module is responsible for catalyzing the post-translational modification at multiple positions of histone H3 including H3K9, H3K18, H3K23, H3K27, H3K36, through the transfer of acetyl groups to the free amino groups of lysine residues (Brownell et al., 1996; Kuo et al., 1996; Grant et al., 1999; Morris et al., 2007; Espinola-Lopez and Tan, 2021). The HAT activity of Gcn5, Ada2, and Ada3 were investigated in different plant pathogens. Knockout of any gene resulted in a significant decrease in the ability to acetylate histone H3 (Lan et al., 2016; Rösler et al., 2016; Chen et al., 2018). Although there is a lack of research on the ability of Ada1 to acetylate histone H3, considering the indispensable role of Ada1 in SAGA, we speculated that after knocking out Ada1, HAT activity of *V. dahliae* would also be affected. Nucleosome acetylation assays in yeast indicated that extracts from Δ*Ada1* cells specifically lacked the nucleosomal HAT activity of the SAGA (Sterner et al., 1999). We tested the impact of Ada1 on HAT activity by western blot analyses and showed that *VdAda1* is critical for acetylation of H3K9, H3K18, and H3K27 in *V. dahliae* (Figure 5A). Furthermore, the expression of other SAGA subunits was affected in Δ*VdAda1* mutant (Figure 5B). As this may be the compensatory effect of the loss of *VdAda1*, it is consistent with previous research confirming that multiple SAGA subunits require Ada1 for the integrity of the SAGA (Wu and Winston, 2002; Wu et al., 2004). In summary, we hypothesized that when *VdAda1* was deleted, the expression of other SAGA subunits could be affected including the HAT module, leading to a decrease in the ability to acetylate histone.

Prior studies suggested that SAGA makes contributions to the expression at ∼10% of the genome in *S. cerevisiae* (Huisinga and Pugh, 2004). However, recent studies have reassessed the role of the SAGA and demonstrated that SAGA is a general cofactor for PIC recruitment that is required for transcription of nearly all genes (Bonnet et al., 2014; Baptista et al., 2017; Warfield et al., 2017; Donczew et al., 2020). In *V. dahliae*, diverse transcription factors regulate fungal vegetative growth and virulence. For example, *VdSge1* (Santhanam and Thomma, 2013), *VdMyo5* (Feng et al., 2018) are required for vegetative growth; *VdCrz1* (Xiong et al., 2015) and *VdCmr1* (Wang et al., 2018) play important roles in microsclerotia development and melanin production, respectively; *VdBre1* (Wang et al., 2021), *VdMcm1* (Xiong et al., 2016) are involved in secondary metabolite (SM) biosynthesis; *VdSNF1*, *Vta2*, *SOM1* together with *Vta3* (Bui et al., 2019) are necessary for successful penetration of host plants. In addition, the pathogen secreted proteins, comprising multiple pathogenic factors, play crucial roles in protecting pathogens from the deleterious effects of host defenses and facilitating colonization of the host plant (Stergiopoulos and de Wit, 2009; Klosterman et al., 2011; Chen et al., 2016), including the LysM effectors (Kombrink et al., 2017), NLP family (Zhou et al., 2012; Santhanam et al., 2013), and small cysteine-rich proteins (SCRPs) (Wang D. et al., 2020). *V. dahliae* avirulence effectors *VdAve1* (de Jonge et al., 2012) and *AV2* (Chavarro-Carrero et al., 2021) activate race-specific resistance. The RNA-Seq analysis revealed a global regulatory effect of *VdAda1* on gene transcription, especially on transcription factors and secreted proteins (Figure 6), thereby affecting multiple physiological processes including fungal growth, nutrient utilization, and virulence (Supplementary Figures 3, 4). As a result, deletion of *VdAda1* resulted in severe phenotypic defects (Supplementary Figures 2, 3).

Pathogenicity assessment showed that *VdAda1*-deleted mutant lost the virulence to cotton and tobacco (Figure 4). This should be a comprehensive result of many aspects. The deletion of *VdAda1* destroys the integrity of the SAGA complex and the function of other module members, resulting in global disorder of transcription and biological processes. Specifically, the absence of *VdAda1* leads to growth retardation and inability to conidiation, which is a prerequisite for infection and colonization by *V. dahliae* (Figure 2). The extremely weak HAT activity causes the damage of histone acetylation modification (Figure 5), and further interferes with the transcription of genes dependent on the SAGA complex, especially the transcription factors and secreted proteins (Figure 6). These components dominated by *VdAda1* may be necessary for virulence of *V. dahliae*.

In summary, the components of the SAGA complex in *V. dahliae* were identified, and VdAda1, as a member of core module of the SAGA, was demonstrated to regulate a number of pathways, most likely through the acetylation of histone. As a consequence, the VdAda1 mutant was defective in vegetative growth, production of conidia, melanin synthesis, and virulence.

## Materials and Methods

### Growth and Melanin Assays

The *V. dahliae* isolate in this study (AT13) was isolated from *Acer truncatum* Bunge in Shandong province, China. This isolate and its transformants were stored at –80°C in 25% glycerol and were cultured on potato dextrose agar (PDA) medium (potato, 200 g/L; glucose, 20 g/L; agar, 15 g/L) at 25°C or in a shaking incubator in complete medium (CM) (yeast extract, 6 g/L; casein acids hydrolyzate, 6 g/ml; sucrose, 10 g/L) at 25°C.

For phenotypic analysis, the equal-sized agar plugs from the wild type, deletion mutant, and the complemented strain colonies were placed in the center of 6-cm-diameter Czapek plates (NaNO_3_, 3 g/L; K_2_HPO_4_, 1 g/L; MgSO_4_⋅7H_2_O, 0.5 g/L; KCl, 0.5 g/L; FeSO4, 0.01 g/L; sucrose, 30 g/L or starch, 17 g/L or pectin, 10 g/L or CMC-Na, 10 g/L; agar, 18 g/L) containing different carbon sources. The colony diameter was measured after 7 days of incubation at 25°C. To evaluate the conidial production, three agar plugs were collected from the edge of the colonies on Czapek plates after 5 days incubation using a 7-mm-diameter hole puncher, and conidia were counted by a hemocytometer after the agar plugs had been shaken in 3 mL of sterile water.

To examine the sensitivity of Δ*VdAda1* to different cell wall stresses, agar plugs of similar sizes from the wild type, deletion mutant, and the complemented strain colonies were placed on PDA medium containing 1 M sorbitol, 200 μg/ml Congo red, and 0.02% SDS. After 7 days of incubation at 25°C, the colony diameters were measured and the inhibition ratio was calculated. Inhibition ratio (%) = (colony diameter on PDA medium – colony diameter on medium containing stressors)/colony diameter on PDA medium × 100%.

To investigate the effect of *VdAda1* on melanin synthesis in *V. dahliae*, agar plugs of similar size from the colonies of the wild type, deletion mutants, and the complemented strains were incubated on PDA and V8 media at 25°C for 2 weeks. In addition, the conidial suspensions from the wild type and complemented strains together with protoplasts from the deletion mutants were adjusted to 1 × 10^6^ propagules/ml and plated on a cellophane membrane (Solarbio, Beijing, China) overlaid on basal agar modified medium (BMM) (Hu et al., 2013). After 2 weeks of incubation at 25°C, microsclerotia on the cellophane layers were photographed.

### Microscopy Observations

To compare hyphae and conidia of the wild type, Δ*VdAda1*, and EC_*VdAda1* strains, the strains were plated on PDA medium and hydrophobic cover slips were inserted around the colony. After the cover slips were removed at 5 dpi, the morphology of hyphae and the production of conidia ofΔ*VdAda1*, wild type and EC_*VdAda1* strains were observed and photographed under differential interference contrast microscopy (#DM6 B & DMC6200, Leica, Germany).

To assess the ability of Δ*VdAda1*, wild type, and EC_*VdAda1* strains to produce microsclerotia, each were cultured in a shaking incubator in liquid complete medium (CM) at 25°C for 3 days. The protoplasts of deletion mutants were cultured in a shaking incubator in liquid TB3 medium (sucrose, 200 g/L; yeast extract, 10 g/L; casamino acid, 10 g/L; glucose, 10 g/L) at 25°C for 3 days. Fungal protoplast preparation of Δ*VdAda1* was conducted according to the method described by Li et al. (2019). The conidial suspensions of wild type and EC_*VdAda1* strain together with protoplasts of Δ*VdAda1* strain were adjusted to 10^6^ conidia or propagules/ml and coated on a cellophane membrane that was overlaid onto BMM medium in plates. After culturing at 25°C for 2 weeks, the morphology of microsclerotia on the cellophane membrane was observed under a stereomicroscope and photographed (#SMZ18, Nikon, Japan).

### Identification of the *V. dahliae* Spt-Ada-Gcn5 Acetyltransferase Complex

To identify putative compositions of the SAGA complex in *V. dahliae* isolate AT13, the *V. dahliae* genome sequence was searched with the BlastP algorithm on the JGI website using protein sequences of known members of the SAGA complex in *S. cerevisiae* S288C as queries. The Sgf11 and Ada1 homologs were identified in *F. fujikuroi*. The Ada1 homolog was also identified in *A. fumigatus* and *V. nonalfalfae*. The Protein domains of all the putative *V. dahliae* SAGA subunits were examined using the Pfam 35.0 network.

### Vector Construction and Fungal Transformation

To generate the *VdAda1* deletion vector, approximately 1 kb upstream and downstream fragments of *VdAda1* were amplified from AT13 genomic DNA. The plasmid pDHt2 (Zhou et al., 2013) was linearized by restriction endonuclease *EcoR*I or *Xba*I, and the amplified fragments were attached before and after the hygromycin resistance gene cassette (*hyg*) by homologous recombination (ClonExpress^®^ II One Step Cloning Kit, Vazyme, Nanjing, China). After the recombinant plasmids were transferred into *Escherichia coli* DH5α competent cells, the deletion vectors were transformed into *Agrobacterium tumefaciens* strain AGL-1, using the *A. tumefaciens* mediated transformation (ATMT) described by Wang et al. (2016). The transformants were selected on PDA infused with hygromycin at 50 μg/mL. The deletion vector pDHt2 encodes a geneticin (G418) resistance gene, so the randomly inserted transformants can grow on medium containing 50 μg/mL geneticin, thus that can exclude the randomly inserted transformants.

The *VdAda1* complemented transformants were produced by polyethylene glycol-mediated protoplast transformation because of the defect of Δ*VdAda1* in conidia production. To generate the *VdAda1* complementation vector, the coding sequence for *VdAda1* with its native promoter and terminator were amplified from AT13 genome and cloned into vector pCOM that carried the geneticin (G418) resistance, and the vector was introduced into the Δ*VdAda1* strain by polyethylene glycol-mediated protoplast transformation. Fungal protoplast preparation of Δ*VdAda1* was conducted according to the method described by Li et al. (2019). For protoplast transformation, 200 μl of 5 × 10^6^ protoplast/ml suspension was added to 5 ng plasmid mixed with an equal volume of 2 × STC [sucrose, 0.4 g/ml; 1M Tris-Cl (pH 8.0), 0.1 ml/ml; CaCl_2_, 0.0147 g] and incubated for 10 min. This protoplast suspension was mixed with 1 × PEG (4 g PEG 4000/1 ml distilled water mixed with an equal volume of 2 × STC) and incubated for 20 min. To this mixture, 3 ml of liquid TB3 medium was added and shaken at 60 rpm for 6 h, 25°C; Cultures were added into 40 ml melted TB3 solid medium (liquid TB3 medium with 8g/L agar) with 50 μg/ml geneticin for selection.

### Penetration and Virulence Assays

For penetration assays, hyphae of each strain were incubated on minimal medium (MM) overlaid with a sterilized cellophane membrane for 3 days. After removal of the cellophane membrane, the cultures were incubated for another 5 days. The experiments were repeated independently at least three times.

Since Δ*VdAda1* is unable to produce conidia, the virulence assay was carried out by inoculating agar plugs from each strain. To prepare for inoculation, the wild type strain AT13, deletion mutants and complementary transformants were cultured on PDA medium for 5 days at 25°C. Cotton (*Gossypium hirsutum* cv. Junmian 1) and *N. benthamiana* were grown in a greenhouse (14 h:10 h, day: night photoperiod) at 25°C ahead of the cultures. For inoculation with *V. dahliae*, a wound at the junction of the root and stem was made using a knife on 2-week-old cotton seedlings or 5-week-old *N. benthamiana* plants and were inoculated with agar plugs collected from the edge of the colonies from each strain. Uninoculated agar plugs served as negative control. Verticillium wilt symptoms were observed 14 days post inoculation on cotton and 21 days post inoculation on *N. benthamiana*. Fifteen cotton plants and five *N. benthamiana* plants were inoculated with each strain. Vascular discoloration of cotton was observed in longitudinal sections of the shoots at 14 dpi. The experiments were repeated independently at least three times.

Biomass of *V. dahliae* was estimated by qPCR in cotton and *N. benthamiana* infected with AT13, Δ*VdAda1*, and complemented strains to investigate the influence of *VdAda1* on colonization of plants. DNA was extracted from the junction of the roots and stems of cotton and *N. benthamiana* inoculated with each strain using DNA secure Plant Kit (TIANGEN, Beijing, China). DNA was quantified by spectrophotometry and 100 ng of DNA of each sample was used for qPCR reaction. A qPCR reaction was performed using genomic DNA as a template and the 2 × TransStart Top Green qPCR SuperMix (TransGen Biotech, Beijing, China) under the following conditions: an initial 95°C denaturation step for 3 min, followed by 40 cycles of 95°C for 15 s, 60°C for 20 s and 72°C for 20 s. *V. dahliae* elongation factor 1-α (*VdEF-1*α) was used to quantify fungal colonization. The cotton *18S* gene and *N. benthamiana EF-1*α were used as endogenous plant reference gene. The primers are listed in supporting information (Supplementary Table 1).

### RNA Extraction and Reverse Transcription-Quantitative PCR

To detect the expression level of melanin-associated genes and SAGA subunits, total RNA was isolated from mycelium of AT13, Δ*VdAda1*, and complemented strains using a total RNA Miniprep kit (Aidlab, Beijing, China). cDNA was reverse transcribed using TransScript II One-Step gDNA Removal and cDNA Synthesis SuperMix (TransGen Biotech, Beijing, China). RT-qPCR was performed under following conditions: an initial 95°C denaturation step for 5 min, followed by 40 cycles of 95°C for 30 s, 60°C for 30 s and 72°C for 20 s. The *V. dahliae VdEF-1*α gene was used as endogenous reference. Relative transcript levels of the above genes in various samples were determined using the 2^–ΔΔCT^ method (Livak and Schmittgen, 2001). Primers used to quantify each gene in RT-qPCR are listed in supporting information (Supplementary Table 1). The experiment was performed three times, and each treatment had three replicates.

For expression pattern analysis of *VdAda1*, wild type strain AT13 was cultured in CM liquid medium for 3 days at 25°C. The roots of cotton seedlings (2-weeks-old) were inoculated with AT13 conidial suspensions (1 × 10^9^ cfu/ml) and roots were harvested at 0, 0.5, 1, 2, 3, 5, 7, and 9 dpi. The RNA was isolated and cDNA was reverse transcribed as described above. The primers used to quantify the expression of *VdAda1* are listed in Supplementary Table 1. The RT-qPCR procedure was carried out as described above.

### RNA-Seq and Gene Expression Profile Analysis

Mycelium from AT13 and Δ*VdAda1* were plated on Czapek medium overlaid with sterilized cellophane membrane and incubated at 25°C for 5 days. The hyphae on cellophane membrane were collected and total RNA was extracted for RNA-seq using total RNA Miniprep kit (Aidlab, Beijing, China). The AT13 strain cultured on Czapek medium was used as a control.

The SOAP-aligner/SOAP2.0 software package (Li et al., 2009) was used to map the reads to the reference sequence of AT13 genome (unpublished), and less than two mismatches were allowed in the alignment. The uniquely mapped read counts were normalized and the expression levels were calculated using the reads per kilobase per million mapped reads (RPKM) method (Mortazavi et al., 2008). A strict algorithm was used to identify significantly differentially expressed genes between the wild type and Δ*VdAda1* strain. The FDR was set to 0.001 to determine the threshold *P* < 0.001 in multiple tests, and the log_2_ ratio ≥2.0 (Audic and Claverie, 1997).

To verify the RNA Seq analysis, the expression level of some of the differentially expressed transcription factors and secreted proteins was examined by RT-qPCR after the total RNA was isolated from mycelium of AT13 and Δ*VdAda1* strains. The RT-qPCR procedure was carried out as described previously. The *V. dahliae* elongation factor 1-α (*VdEF-1*α) was used as a reference gene for the relative expression analysis. The primers used to quantify the expression of these genes were shown in Supplementary Table 1.

### Western Blot Analyses

For Western blot analyses, total protein was extracted from lyophilized mycelium of wild type AT13, Δ*VdAda1*, and complemented strains cultured in CM liquid medium for 3 days. Mycelia (50 mg) were finely ground and suspended in extraction buffer (RIPA lysis buffer: 500 μl; phenylmethylsulfonyl fluoride (PMSF), 1 mM). After homogenization by vortexing, the lysate was centrifuged at 12,000 rpm for 10 min at 4°C. 100 μl supernatant was mixed with an equal volume of 2× loading buffer and boiled for 10 min. Thirty micrograms of protein were separated by SDS PAGE (15%). After transferring to Immobilon-P transfer membranes (Merck Millipore, Darmstadt, Germany), the membranes were probed with anti-H3K9ac (#39138, 1:10000), -H3K18ac (#39756, 1:10000), -H3K27ac (#39136, 1:10000) (Active Motif, La Hulpe, Belgium), anti-H3 (M130918, Hunan Biotechnology Co., Ltd., Hangzhou, Zhejiang, China, 1:5000) primary antibodies and a goat anti-rabbit IgG-HRP secondary antibody (CWBIO, Beijing, China, 1:10000). Chemiluminescence was detected with Immobilon Western Chemoluminescent HRP Substrate (Merck Millipore, Darmstadt, Germany). The experiment was conducted three times independently.

## Data Availability Statement

The datasets presented in this study can be found in online repositories. The names of the repository/repositories and accession number(s) can be found below: https://www.ncbi.nlm.nih.gov/Traces/study/?acc=PRJNA800720&o=acc_s%3Aa.

## Author Contributions

D-DZ, F-MC, and J-YC conceived and designed the experiments. QG, HL, DW, R-CS, and HZ performed the experiments. D-DZ and J-YC analyzed the data and performed bioinformatic analysis. QG and HL prepared biological material. D-DZ and QG wrote the original draft. KS, SK, and J-YC reviewed and edited the manuscript. All authors contributed to the article and approved the submitted version.

## Conflict of Interest

The authors declare that the research was conducted in the absence of any commercial or financial relationships that could be construed as a potential conflict of interest.

## Publisher’s Note

All claims expressed in this article are solely those of the authors and do not necessarily represent those of their affiliated organizations, or those of the publisher, the editors and the reviewers. Any product that may be evaluated in this article, or claim that may be made by its manufacturer, is not guaranteed or endorsed by the publisher.
