# Supplementary material for: The Verticillium dahliae Spt-Ada-Gcn5 Acetyltransferase Complex Subunit Ada1 Is Essential for Conidia and Microsclerotia Production and Contributes to Virulence
Source: Front Microbiol. 2022 Feb 23;13:852571. doi: 10.3389/fmicb.2022.852571 (PMC8905346; doi:10.3389/fmicb.2022.852571)
Supplement: Supplementary file 1 [file Data_Sheet_1.docx]

**Supporting information**

**
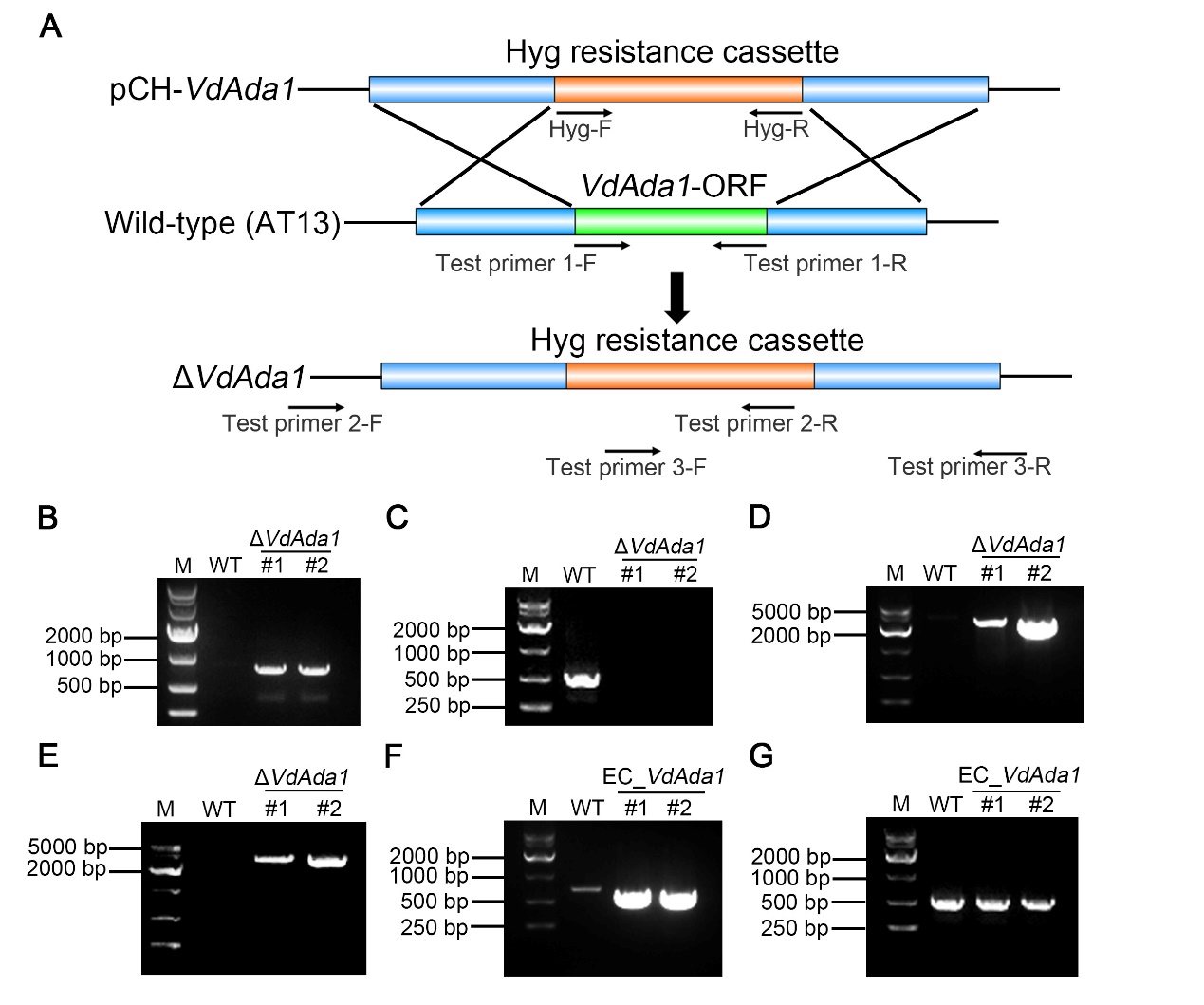
**

**Fig. S1.** Construction and genetic identification of *VdAda1* deletion mutants and complemented transformants. (A) Pictorial representation of the procedure followed for construction of *VdAda1* deletion mutants, and primers used for testing mutants. The deletion vector pDHt2 carries the geneticin (G418) resistance, thus can screen out randomly inserted transformants. Hygromycin resistance gene (Hyg) cassette (*hyg*) replaced *VdAda1* by homologous recombination. (B) Genetic identification of two independent deletion mutants with the primers Hyg-F and Hyg-R by PCR. (C) Genetic identification of the deletion mutants with the primers Test primer 1-F and Test primer 1-R by PCR, which were designed in the *VdAda1*-orf. (D) PCR identification of the deletion mutants with the primers Test primer 2-F and Test primer 2-R. (E) PCR identification of the deletion mutants with the primers Test primer 3-F and Test primer 3-R. (F) The full-length of *VdAda1* gene was cloned into the binary vector pCOM that carried the geneticin (G418) resistance, and the vector was introduced into Δ*VdAda1* mutant by homologous recombination to produce complementation transformants EC_*VdAda1*. Geneticin (G418) resistance was detected in two independent complemented transformants by PCR. (G) Genetic identification of the complemented mutants with the primers Test primer 1-F and Test primer 1-R by PCR, which were designed to amplify a fragment within *VdAda1*.

**
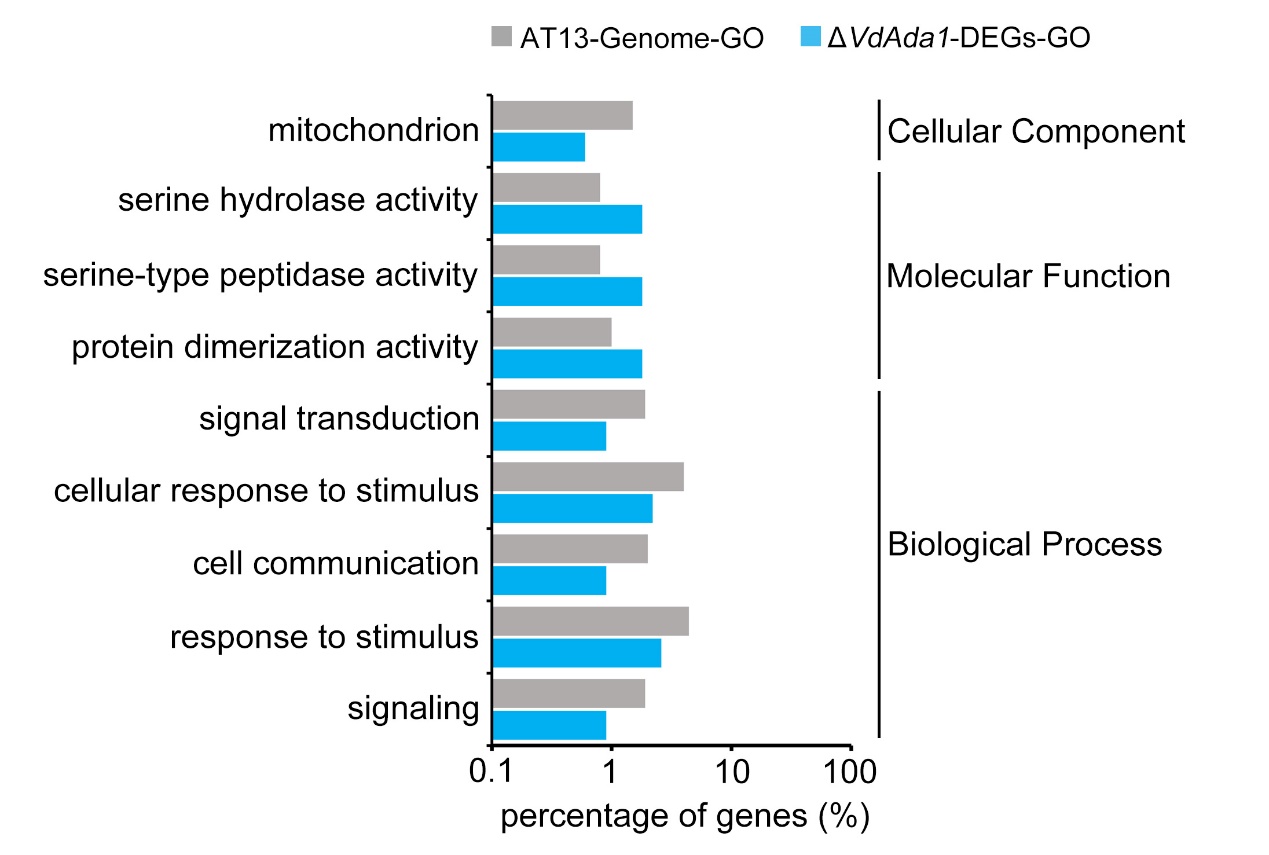
**

**Fig. S2** Gene ontology (GO) annotation of AT13 genome and DEGs in Δ*VdAda1* strain compared with AT13. Functional clustering was performed using WEGO software.­


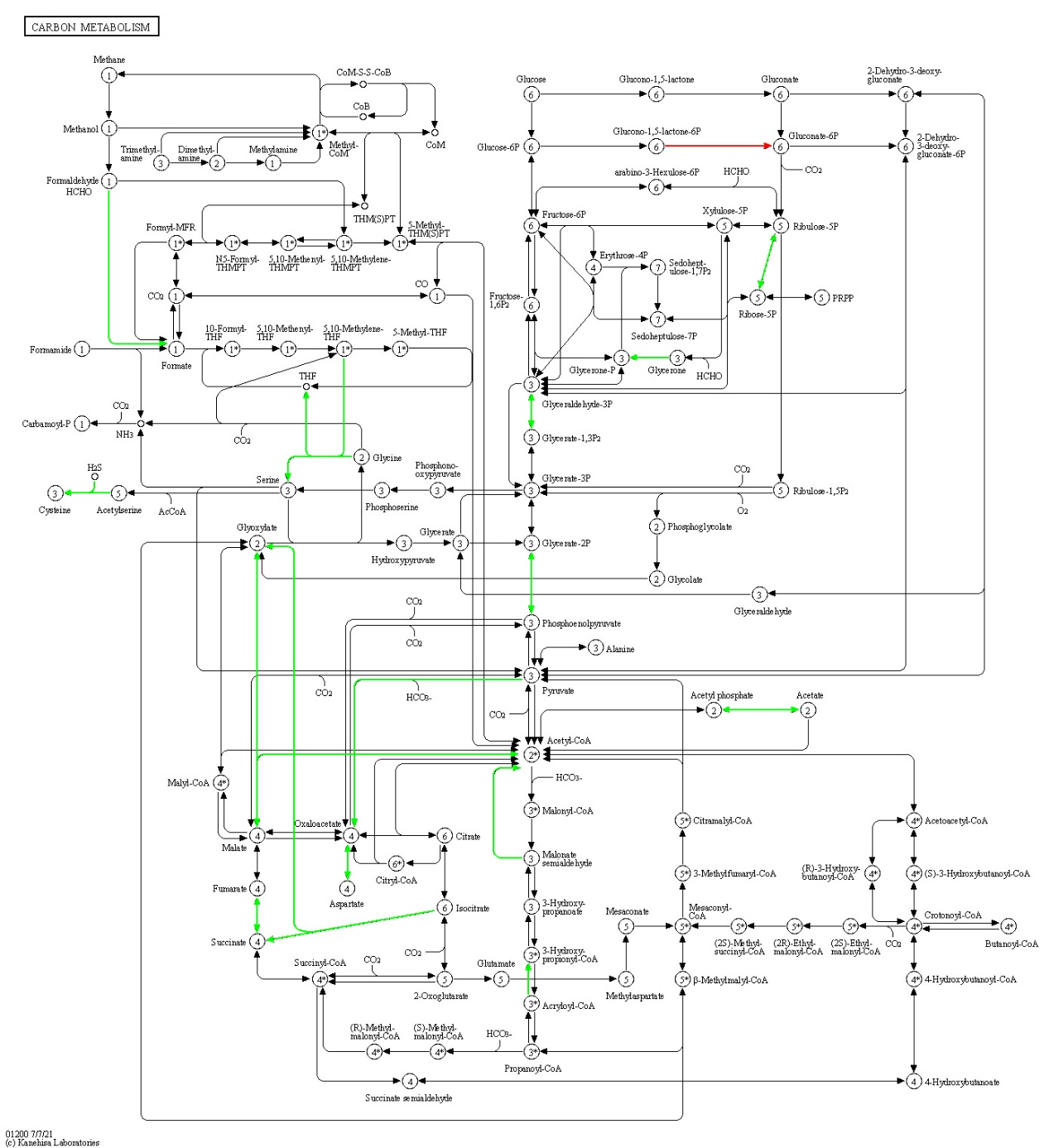


**Fig. S3** *VdAda1* deletion effects in *V. dahliae*. DEGs were matched to carbon metabolism (ko:01200) using the KEGG database. Steps which are enriched for genes downregulated in Δ*VdAda1* strain are highlighted in green color, while steps enriched for genes upregulated in Δ*VdAda1* strain are highlighted in red color.


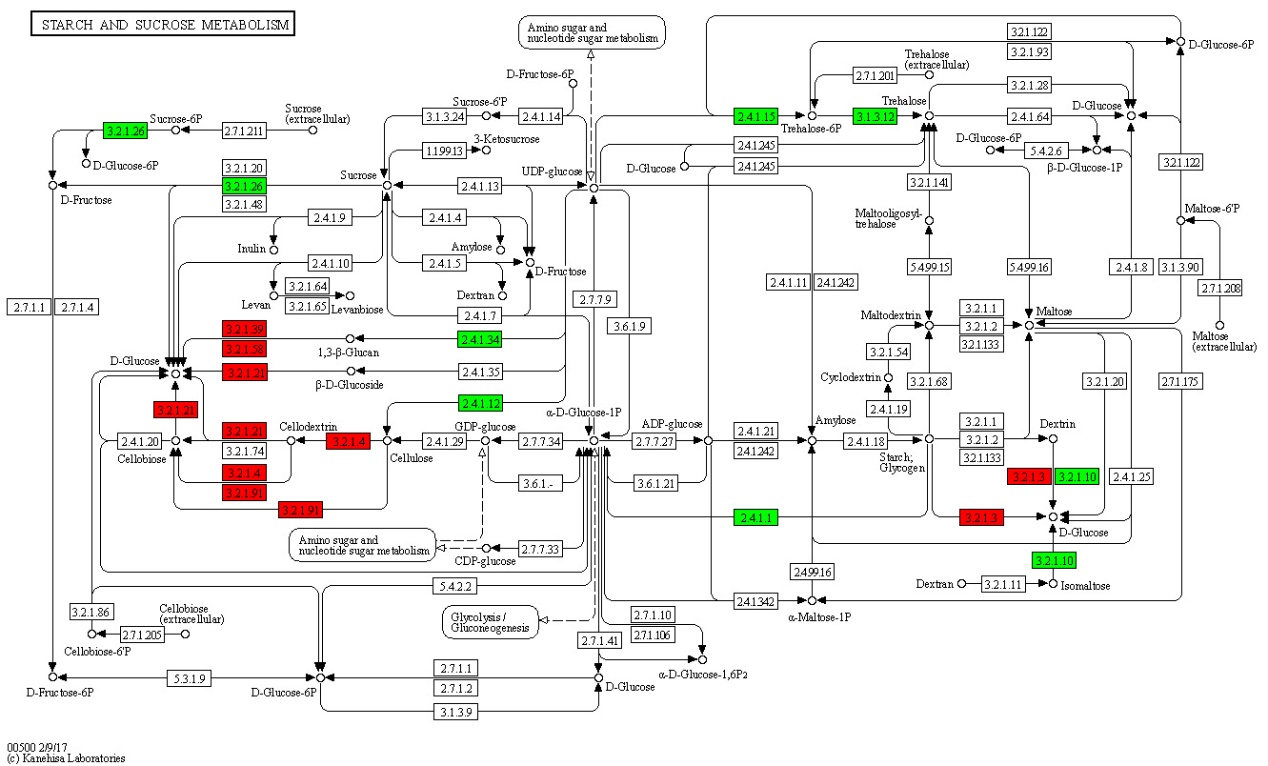


**Fig. S4** Identification of genes involved in starch and sucrose metabolism. DEGs were matched to starch and sucrose metabolism pathways (ko:00500) using the KEGG database. The genes downregulated in Δ*VdAda1* strain are highlighted in green color, while the genes upregulated in Δ*VdAda1* strain are highlighted in red color.
